# Determinants of Serum Glycerophospholipid Fatty Acids in Cystic Fibrosis

**DOI:** 10.3390/ijms18010185

**Published:** 2017-01-18

**Authors:** Sławomira Drzymała-Czyż, Patrycja Krzyżanowska, Berthold Koletzko, Jan Nowak, Anna Miśkiewicz-Chotnicka, Jerzy A. Moczko, Aleksandra Lisowska, Jarosław Walkowiak

**Affiliations:** 1 Department of Pediatric Gastroenterology and Metabolic Diseases, Poznan University of Medical Sciences, Szpitalna 27/33, 60-572 Poznań, Poland; p.krzyzanowska@tlen.pl (P.K.); j-nowak@outlook.com (J.N.); mucha604@interia.pl (A.M.-C.); alisow1@wp.pl (A.L.); jarwalk@ump.edu.pl (J.W.); 2 Ludwig-Maximilians-Universität München, Division of Metabolic and Nutritional Medicine, Dr. von Hauner Children’s Hospital, University of Munich Medical Center, Lindwurmstr. 4, D-80337 Munich, Germany; office.koletzko@med.lmu.de; 3 Department of Computer Science and Statistics, Poznan University of Medical Sciences, Dąbrowskiego 79, 60-529 Poznań, Poland; jmoczko@ump.edu.pl

**Keywords:** phospholipids, polyunsaturated fatty acid, docosahexaenoic acid, eicosapentaenoic acid, arachidonic acid

## Abstract

The etiology of altered blood fatty acid (FA) composition in cystic fibrosis (CF) is understood only partially. We aimed to investigate the determinants of serum glycerophospholipids’ FAs in CF with regard to the highest number of FAs and in the largest cohort to date. The study comprised 172 CF patients and 30 healthy subjects (HS). We assessed Fas’ profile (gas chromatography/mass spectrometry), CF transmembrane conductance regulator (*CFTR*) genotype, spirometry, fecal elastase-1, body height and weight *Z-scores*, liver disease, diabetes and colonization by *Pseudomonas aeruginosa*. The amounts of saturated FAs (C14:0, C16:0) and monounsaturated FAs (C16:1n-7, C18:1n-9, C20:1n-9, C20:3n-9) were significantly higher in CF patients than in HS. C18:3n-6, C20:3n-6 and C22:4n-6 levels were also higher in CF, but C18:2n-6, C20:2n-6 and C20:4n-6, as well as C22:6n-3, were lower. In a multiple regression analysis, levels of seven FAs were predicted by various sets of factors that included age, genotype, forced expiratory volume in one second, pancreatic status and diabetes. FA composition abnormalities are highly prevalent in CF patients. They seem to be caused by both metabolic disturbances and independent clinical risk factors. Further research into the influence of *CFTR* mutations on fat metabolism and desaturases’ activity is warranted.

## 1. Introduction and Aims

The fatty acid (FA) profiles of serum and blood cells influence a range of metabolic, immune, and other functions [1]. An individual’s diet and metabolic characteristics determine the FAs’ availability to the blood and also to other tissues [2,3,4,5]. Many studies have documented that the n-3 long-chain polyunsaturated fatty acid (LC-PUFA) supplementation has positive effects on inflammation and immunity [6,7]. The FA status of patients can be affected by diet (e.g., the consumption of fatty fish and their oils) or by disease-related changes in FA absorption or metabolism, which is the case in cystic fibrosis (CF) [2,8].

CF is the most common genetic disorder in the Caucasian population. It is caused by a dysfunction of the CF transmembrane conductance regulator (CFTR) [9]. The consequences of CFTR-related disruption of transmembrane ion transport are manifested in a number of organ systems and lead to intestinal, pancreatic, and hepatobiliary complications [10]. *CFTR* mutations do not uniformly translate to CFTR dysfunction and/or organ damage; the reasons for this are complex and only partially understood. One of the potential factors affecting the genotype–phenotype relationship is FA metabolism. In 1962, Kuo et al. [11] found changes in FA composition in blood and tissues of CF patients for the first time. Since then, many groups have described abnormalities in the FA profile, which include an increased level of saturated fatty acids and also C16:1n-7 (palmitoleic acid, POA) and C20:3n-9 (mead acid). Decreased amounts of unsaturated FAs were also described: C18:2n-6 (linoleic acid; LA), C20:4n-6 (arachidonic acid; AA) and C22:6n-3 (docosahexaenoic acid; DHA) [8,12,13,14,15,16,17,18,19,20,21,22]. Despite previous investigations, the exact causes of these abnormalities are unknown and their disentanglement constitutes a current challenge in CF research [23].

The aims of this study were to assess the FA composition of serum glycerophospholipids in CF patients and to investigate its exogenous and endogenous determinants. This research was planned to further our understanding of the topic by two main means: (1) inclusion of the largest cohort (that actually involved 10% of all Polish CF patients); and (2) measurement of the largest number of FAs in CF to date.

## 2. Results

The anthropometric and clinical parameters describing CF patients are presented in Table 1. Weight and height *Z-score* values lower than −2 were found in 23 and 28 patients, respectively. Elevated alanine aminotransferase (ALT), aspartate aminotransferase (AST) and **γ**-glutamyltransferase (GGT) values were found in 36, 28 and 29 patients, respectively. Significant impairment in forced expiratory volume (FEV1) (<40%) was found in 30 patients; 55 patients had good lung function (>80%).

Twenty-four patients were pancreatic-sufficient, while the remaining 148 subjects required pancreatic enzyme supplementation. Liver disease was found in 79 patients. Diabetes was documented in 25 patients. Eighty-eight patients were colonized with *Pseudomonas aeruginosa* (*P. aeruginosa*); the colonization with *Staphylococcus aureus* (*S. aureus*) or *Burkholderia cepacia* (*B. cepacia*) was found in 18 and four patients, respectively.

The CF patients’ adherence to nutritional recommendations was poor [24]. The energy intake was lower than the 120%–150% of the recommended daily amount (independent of age, malnutrition or concurrent disease). However, the energy distribution of protein and fat was adequate (Table 2). The daily intake of C18:2n-6 (LA) and C18:3n-3 (α-linolenic acid; ALA) was insufficient in 50% (less than 4% of total energy) and 29% (less than 0.50% of total energy) of patients, respectively [25,26].

The profile of selected FAs in serum glycerophospholipids of the CF patients is shown in Table 3. A subgroup of adult CF patients (*n* = 32) was created, whose age matched that of healthy subjects (HS) most closely (median [first–third quartile]: 23.5 [20.8–30.3] vs. 22.3 [20.4–23.4] years, ns.). The amounts of saturated FAs (SFAs; C14:0, C16:0) and monounsaturated FAs (MUFAs; C16:1n-7, C18:1n-9, C20:1n-9, C20:3n-9) were significantly higher in CF patients than in HS.

C18:2n-6 (LA), C20:2n-6 (eicosadienoic acid) and C20:4n-6 (AA) were lower in the CF group than in HS. However, C18:3n-6, C20:3n-6 (dihomo-γ-linolenic acid) and C22:4n-6 were higher in CF patients. Among all analyzed n-3 polyunsaturated FAs, only C22:6n-3 (DHA) was significantly lower in the CF group compared with HS. The n-6/n-3 ratio was higher in CF patients than in HS. All of the above differences were significant also in the comparison of the age-matched subgroup of adult CF patients with HS, but one (C20:3n-6).

The C18:3n-6/C18:2n-6 ratio (reaction catalyzed by Δ6-desaturase) was significantly higher in CF patients compared with HS. On the other hand, the C20:4n-6/C20:3n-6 ratio (Δ5-desaturase), C22:5n-6/C22:4n-6 and C22:6n-3/C22:5n-3 ratios (both latter reactions catalyzed by Δ6-desaturase) were lower in CF patients than in HS. Interestingly, the ratio between the last and the first FA in n-3 (C22:6n-3/C18:3n-3) was lower in CF patients than in HS.

The FA profile of serum glycerophospholipids in the CF patients depending on the different clinical parameters is presented in Table 4. Among the many differences identified, which are also indicated in the table, we would like to highlight those pertaining to three FAs. C16:1n-7 was higher in pancreatic insufficiency, liver disease and diabetes. C20:3n-9 levels were higher in patients with a severe *CFTR* genotype, exocrine pancreatic insufficiency, insufficient height and also in children. C20:4n-6 was lower in CF patients with severe genotypes, liver disease, low weight and height *Z-scores* and diabetes. C22:5n-3 was lower in patients with the lowest *Z-scores* of body weight, liver disease, diabetes and colonization of *P. aeruginosa*.

A multiple regression analysis identified variables predicting C16:0, C18:2n-6, C18:3n-6, C20:3n-6, C20:4n-6, C22:5n-6 and C22:6n-3 status. The results of the multiple regression models are summarized in Table 5.

## 3. Discussion

The FA composition of serum glycerophospholipids is a valuable biomarker of FA status in humans [3,27]. The main putative causes of FAs’ abnormalities in CF patients include diet, pancreatic insufficiency, liver disease and diabetes. However, the observational data gathered thus far does not fit those mechanistic predictions sufficiently to establish hypotheses of causative relationships [12,13,14,16,28,29]. Our study applied a multiple regression analysis to the largest cohort of CF patients, in whom the largest number of FAs to date was measured. It provides new insights into the determinants of glycerophospholipids FAs’ abnormalities in CF and the pathology of FAs’ metabolism in CF, which are discussed below. The obtained dataset, which is the largest to date, is herein made publicly available to the community with the hope that it will help further the understanding of CF-related disturbances in the FA metabolism.

This research identified significant abnormalities in the FAs’ profile in CF patients. The three biologically most important essential fatty acids (EFA)—LA, AA and DHA—were reduced in spite of dietary EFA intakes that were in line with recommended intakes [25,26], which points to a possible role of reduced bioavailability due to fat malassimilation and/or increased turnover. The higher rate of lipid turnover in cell membranes in CF was described by Rogiers et al. [30], who did not prove the influence of extrinsic factors, or explain the mechanism otherwise. A reduced LA level has been repeatedly found in CF in both blood (serum, plasma, erythrocytes) [12,13,14,17,28,29] and in the adipose tissue, skeletal and cardiac muscle, liver, lungs, and nasal epithelium [11,14]. The available data show that the LA level was significantly lower in patients with severe *CFTR* mutations, with worse pulmonary function (lower FEV1 percentage) and pancreatic insufficiency [12,13,15].

Many studies showed that the low AA status depends on the accelerated metabolism of this FA, that increased expression and activity of Δ5- and Δ6-desaturases is associated with severe mutations in the *CFTR* gene and also that this was more frequent in patients with pancreatic insufficiency and worse pulmonary function [12,13,14,23,31,32]. Increased Δ6-desaturase activity could explain the elevated C18:3n-6/C18:2n-6 ratio found in our study, whereas increased PUFA conversion has little effect on the final metabolites’ DHA and AA [33]. It should be underscored that the low AA level may also result from its increased use during inflammation. We took note of the lower amount of this FA in patients with *Z-scores* for body weight and height ≤−2, with two severe mutations of the *CFTR* gene, with liver disease and diabetes. Our findings concerning the changes in n-6 FA status can result from an increased n-6 turnover (a higher C22:5n-6/C18:2n-6 ratio was found in adult CF patients). They also suggest that EFA abnormalities correlate with disease severity.

In some studies, the ALA and C22:5n-3 (docosapentaenoic acid n-3; DPAn-3) content was found to be reduced [34] but in other reports it was found either unchanged [29,35] or even increased [18,35]. Such variability in results could be explained by a change of PUFA metabolism and it could also result from reduced EFA bioavailability (malnutrition, pancreatic insufficiency, presence of comorbidities, such as liver disease) [36]. In our study, the level of DPAn-3 was lower in patients with comorbidities as well.

In some other studies, C20:5n-3 did not differ significantly from the controls [13,35,37]; in others, it was increased [21,38], or decreased [22,34,39]. Reduced DHA is one of the most common changes seen in CF (both in cell culture and in human studies) [5,12,13,14,16,28,29,35,40]. In previous studies, C22:5n-3 concentration reductions correlated with more severe *CFTR* mutations, worse pulmonary function and CF related liver disease [12,15,41]. This is partially supported by our research—we found reduced C22:5n-3/C22:6n-3 and C22:6n-3/C18:3n-3 ratios in comparisons of the FA profile in subgroups of CF patients defined by different clinical parameters.

There are multiple possible explanations for DHA deficiency in CF. Firstly, the coexistence of normal or increased levels of EPA with decreased DHA could be caused by a low conversion of EPA to DHA [21] or by a retroconversion of DHA to EPA [42,43]. This reversal was shown to be typical of n-3 FA metabolism, which occurs through modified β-oxidation in peroxisomes [42,43]. Secondly, because of a CF-associated defect in the methyl group metabolism, a larger share of phosphatidylcholine is produced de novo than from phosphatidylethanolamine. The former pathway incorporates less DHA [44,45]. Third, the low DHA could also result from DHA catabolism, a consequence of DHA being the precursor of such mediators as resolvins, maresins and docosatrienes, which are required for the resolution of inflammation in CF patients [46,47]. Although the regression analysis also pointed towards pancreatic insufficiency and *CFTR* genotypes as independent correlates of low DHA levels, our work does not identify these two factors as the main determinants of serum FA alterations in CF.

Increased levels of C18:3n-6 (γ-linolenic acid; GLA) were also reported by Lloyd-Still et al. [34] in CF infants, older children and adults. The multiple regression analysis of other n-6 FAs in our study showed that age, diabetes, *CFTR* genotype, liver disease and pancreatic insufficiency were all significant predictors of n-6 FAs’ abnormalities (GLA, C20:3n-6, C20:4n-6, C22:4n-6). C20:2n-6, C22:4n-6, C22:5n-6, which are not included in the routine assessment of FAs’ composition, were measured in our study. Interestingly, when n-6 FAs’ metabolism is considered globally, an alternating pattern is seen (low C18:2n-6, high C18:3n-6, low C20:2n-6, high C20:3n-6, low C20:4n-6). This could possibly indicate an imbalance between desaturase activity and utilization of FAs.

Another major disturbance in FA composition in our CF patients is the increased level of SFAs. The changes in SFAs’ status were previously described by Olveira et al. [13]. They found that the percentages of myristic and stearic acids were significantly higher in the CF group than in control subjects. However, there was no difference between SFA levels with regard to pancreatic insufficiency, *CFTR* genotype, FEV1 or malnutrition. It should be underscored that this research involved only 37 patients (which imposes limitations on interpretation) and that the authors carried out a univariate analysis. It is difficult to explain which factors determine the observed disturbances. Our results suggest that the changes reflect the genotype-dependent severity of CF course and the presence of comorbidities, such as liver disease. Fat malassimilation is expected to increase the ratio of bioavailable carbohydrates to fat, resulting in an increased de novo biosynthesis of SFAs, such as palmitic acid, as previously proposed [48,49].

An increase in C16:1n-7 is one of the most common disturbances occurring in CF patients [12,13,28] and may result from desaturation of endogenously synthesized palmitic acid. However, none of the available studies involved a wider analysis of individual MUFAs. The present study revealed that levels of the four MUFAs were higher in CF patients than in HS. MUFAs can be synthesized from SFAs; this process involves the introduction of a first double bond in the Δ9 position [31,40,50]. Since EFAs’ deficiency induces MUFAs’ integration into phospholipids, serum glycerophospholipids’ mead acid concentration can be used as its marker [50]. CF-associated chronic inflammation contributes to a paucity of C18:2n-6 and leaves the conversion of C18:1n-9 to C20:3n-9 uninfluenced by mechanisms which would stop it in physiological conditions.

In conclusion, we found a high prevalence of FA composition abnormalities in CF patients. The observed changes may be influenced by both metabolic disturbances—as indicate differences in proportions between selected FAs—and independent clinical risk factors: genotype, diabetes, age, FEV1, liver disease and pancreatic insufficiency. Further research into the influence of *CFTR* mutations on fat metabolism and desaturases’ activity is warranted.

## 4. Materials and Methods

### 4.1. Patients

The study comprised of 172 CF patients: 86 female, 86 male; aged 4 to 50 years; 77 children and 95 adults. The inclusion criteria were as follows: CF was diagnosed according to current guidelines [51], based on clinical presentation, sweat test results and genetic testing. The exclusion criteria were age below 4 years, pregnancy and lung transplantation. The control group consisted of 30 healthy subjects: 20 female, 10 male, aged 18 to 25 years; body mass index (BMI)—median [interquartile range]: 21.04 [19.96–22.23] kg/m^2^. Individual CF characteristics were assessed: *Z-score* for body height and weight [52]; lung function—spirometry (FEV1 (%)), it was determined in subjects older than 6 years; exocrine pancreatic function—fecal elastase-1 [53,54]; liver disease defined as liver pathology diagnosed in the past, confirmed by three repetitive, increased activities of liver enzymes—ALT and AST (any time beyond first year of life) and increased/heterogenous liver echogenicity on ultrasonography [55]; colonization by *P. aeruginosa*, *S. aureus* and *B. cepacia*—chronic and/or intermittent; diabetes, which was diagnosed (or diagnosis confirmed) according to International Society for Pediatric and Adolescent Diabetes Clinical Practice Consensus Guidelines 2014 [56]; *CFTR* genotype—mutations were classified as severe (types I, II, III) and other: mild (IV, V) or unknown.

Mutations in one or both alleles of the *CFTR* gene were identified in 154 patients (89.5%). The genotypes of the studied CF patients were as follows: F508del/F508del (*n* =  75); F508del/- (*n* = 16); F508del/3849  +  10 kbC  >  T (*n* =  10); F508del/2143delT (*n* =  4); F508del/R553X (*n*  =  3); F508del/3272-26A > G (*n* = 3); F508del/2184insA (*n* = 2); F508del/1717-1G > A (*n* = 2); F508del/2183AA  >  G (*n*  =  2); F508del/CFTRdel21 (*n* = 2); F508del/N1303K (*n* =  2); F508del/W1282x (*n* = 2); F508del/2721AATTTGGTGCT (*n* = 1); F508del/3121-2A > G (*n* = 1); F508del/3171insC (*n* = 1); F508del/3600 + 2insT (*n* = 1); F508del/G551D (*n* = 1); F508del/R117H (*n* = 1); F508del/R352Q (*n* = 1); F508del/R851X (*n* = 1); F508del/3659delC (*n* = 1); F508del/C525X (*n* =  1); F508del/c.3718-2477C > T (*n* = 1); 1717-1G > A/CFTRdel2,3(21kb) (*n* = 1); 1717-1G > A/- (*n* = 1); 2143delT/R1102X (*n* = 1); C524X/G524X (*n* = 1); dele2,3(21kb)/3849 + 10kb C > T (*n* = 1); R347P/R347P (*n* = 1); S1196X/Q1382X (*n* = 1); 1524 + 1G > A/3944delGT;406-6T > C (*n* = 1); 2183AA-G/1717-1G- > A (*n* = 1); 3659delC/R153i (*n* = 1); 3849 + 10kbC > T/3600 + 1G > T (*n* = 1); CFTRdele2,3(21kb)/CFTRdele2,3(21kb) (*n* = 1); CFTRdele2,3(21kb)/- (*n* = 1); T581l/2721del11 (*n* = 1); N1303K/CFTRdele2,3(21kb) (*n* = 1); N1303K/G551D (*n* = 1); N1303K/- (*n* = 1); 2184insA/- (*n* = 1); G542X/- (*n* = 1); 3272-26A > A/- (*n* = 1). Patients and HS were involved in the study in the years 2013–2015.

### 4.2. Dietary Intake

The frequency of the consumption of fish, olive oil, other oils and other products rich in FAs was assessed. To estimate energy and macronutrient intake, subjects were asked to complete a 3-day diary (2 working days and 1 day during the weekend). The dietitian (Sławomira Drzymała-Czyż) analyzed the diaries, taking into account the calories deriving from all possible sources of dietary fat. No patient took supplements enriched with FAs (e.g., blubber). For all patients, the total energy requirement was calculated and compared to daily dietary intakes. The percentages of daily energy requirement covered were thus obtained. The analyses were performed with the use of Dietetyk 2015 software (Jumar, Poznań, Poland).

### 4.3. Fatty Acid Analysis

Blood samples were collected after an overnight fasting period. Samples were centrifuged (3000× *g*, 10 min, 4 °C), and stored in plastic vials at −80 °C.

The profile of the FAs was assessed using Glaser’s method [2]. One hundred microliters of serum, 100 μL of an internal standard (146 µg PC15:0/mL methanol), and 0.6 mL of cold methanol were combined in glass tubes and shaken for 30 s. After centrifugation (2300× *g*, 10 min, 4 °C), the supernatant was transferred into a glass vial. FA methyl esters (FAMEs) were synthesized at room temperature by adding 25 µL sodium methoxide (25 wt % in methanol; Sigma Aldrich, Saint Louis, MO, USA). The transesterification reaction was stopped after 3 min by adding 75 µL 3 M methanolic HCl. FAMEs were extracted twice into 2× 300 µL hexane. The extracts were combined and the solvent was evaporated under nitrogen. FAMEs were redissolved in 50 µL hexane containing butylated hydroxytoluene (2 g/L) and stored at −20 °C until further analysis.

Individual FAMEs were quantified by gas chromatography with mass spectrum (Agilent 7890 series II and 5975C, Agilent Technologies, Santa Clara, CA, USA) using a BPX 70 column (BPX70, 25 m × 0.22 mm ID × 0.25 μm, SGE Analytical Science, Ringwood, Australia) as described previously [2]. Peak integration was performed using MSD ChemStation (Agilent Technologies, Santa Clara, CA, USA).

### 4.4. Statistical Methods

The results of the FAs’ analysis were expressed as percentage values (% *wt*/*wt*). For all parameters, medians and first–third quartiles were calculated. The Shapiro-Wilk test was used to check the normality of the data distribution. The Mann-Whitney U-test was used to assess differences between subgroups (Table 3 and Table 4). The influence of all studied parameters on the FAs’ profile was assessed using a multiple linear logistic regression (stepwise and backward). Values of *p* < 0.05 were considered to be statistically significant. All statistical analyses were performed in Statistica 12.0 software environment (StatSoft Inc., Tulsa, OK, USA) and Stata/IC 14.0 for Windows (StataCorp LP, Lakeway Drive, TX, USA). The following variables were interpreted as independent in the regression model: age, *Z-score* for body weight and height, *CFTR* mutation, FEV1, pancreatic insufficiency, liver disease, diabetes and *P. aeruginosa* colonization.

### 4.5. Ethical Considerations

The protocol of the investigation was approved by the Bioethical Committee at Poznań University of Medical Sciences, Poznań, Poland (decision no. 250/10). Written informed consent was obtained from all adult participants and, in the case of children, from their parents. The study was carried out in accordance with the revised Declaration of Helsinki.

## Figures and Tables

**Table 1:** Clinical and demographic data of cystic fibrosis (CF) patients.

Clinical Parameters	Median	1st–3rd Quartile
Age (years)	18.9	12.5–26.9
*Z-score* for body weight	−0.73	−1.46 to −0.10
*Z-score* for body height	−0.71	−1.44–0.10
ALT (U/L)	22.0	15.0–33.0
AST (U/L)	25.0	18.0–32.3
GGT (U/L)	17.0	12.0–29.0
FEV1 (%)	65.0	45.2–90.4

ALT: alanine aminotransferase; AST: aspartate aminotransferase, GGT: **γ**-glutamyltransferase.

**Table 2:** Dietary intakes in cystic fibrosis (CF) patients.

Dietary Intake	Median	1st–3rd Quartile
Energy (kcal/day)	2430	2144–3297
EER * (%)	116.0	97.2–140.7
Protein (% en)	15.6	13.4–17.2
Carbohydrates (% en)	45.3	38.1–50.3
Total fat (% en)	39.4	35.3–45.0
Saturated fat (% en)	15.6	13.0–17.8
Monounsaturated fat (% en)	14.5	12.7–17.6
Polyunsaturated fat (% en)	5.1	4.4–7.0
C18:2n-6 (% en)	3.9	3.1–5.1
C18:3n-3 (% en)	0.6	0.5–0.7
n-6 (% of total fat)	9.9	7.9–13.5
n-3 (% of total fat)	1.5	1.2–2.0
n-6/n-3	6.2	4.8–8.3

* EER: estimated daily energy requirement.

**Table 3:** The profile of selected fatty acids in phospholipids of cystic fibrosis (CF) patients’ and healthy subjects’ (HS) blood serum.

Fatty Acid	All CF *n* = 172	CF Adults *n* = 32	HS *n* = 30
Median, % *wt*/*wt* (1st–3rd quartile)			
**Saturated fatty acids**			
C14:0 (myristic acid)	0.61 ^†^ (0.49–0.78)	0.56 * (0.52–0.74)	0.52 (0.40–0.61)
C16:0 (palmitic acid)	31.10 ^‡^ (29.23–32.70)	30.06 * (28.81–34.24)	28.79 (27.17–30.24)
C18:0 (stearic acid)	16.32 (14.75–18.89)	16.17 (14.89–20.64)	16.20 (14.90–17.73)
**Monounsaturated fatty acids**			
C16:1n-7 (palmitoleic acid)	1.04 ^‡^ (0.76–1.43)	1.18 ^‡^ (0.86–1.85)	0.57 (0.50–0.68)
C18:1n-9 (oleic acid)	13.22 ^†^ (11.97–14.68)	13.04 ^‡^ (12.29–15.33)	11.60 (10.37–13.17)
C20:1n-9 (eicosenoic acid)	0.20 ^‡^ (0.13–0.31)	0.36 ^‡^ (0.20–0.48)	0.11 (0.09–0.18)
C20:3n-9 (mead acid)	0.40 ^‡^ (0.26–0.86)	0.45 ^‡^ (0.32–0.94)	0.20 (0.10–0.40)
**n-6 polyunsaturated fatty acids**			
C18:2n-6 (linoleic acid)	18.63 ^‡^ (16.06–20.51)	16.49 ^‡^ (15.38–20.24)	21.49 (20.38–22.57)
C18:3n-6 (γ-linolenic acid)	0.31 ^‡^ (0.21–0.52)	0.43 ^‡^ (0.36–0.61)	0.17 (0.12–0.21)
C20:2n-6 (eicosadienoic acid)	0.28 ^†^ (0.22–0.34)	0.28 ^†^ (0.21–0.37)	0.33 (0.28–0.40)
C20:3n-6 (dihomo-γ-linolenic acid)	2.97 ^†^ (2.37–3.63)	2.75 (2.09–3.63)	2.63 (2.12–2.91)
C20:4n-6 (arachidonic acid)	7.80 ^‡^ (6.56–8.90)	8.09 * (5.26–9.81)	9.35 (8.62–10.23)
C22:4n-6 (docosatetraenoic acid)	0.30 * (0.22–0.39)	0.32 * (0.23–0.42)	0.26 (0.20–0.29)
C22:5n-6 (docosapentaenoic acid n-6)	0.26 (0.19–0.41)	0.31 (0.18–0.48)	0.27 (0.21–0.34)
**n-3 polyunsaturated fatty acids**			
C18:3n-3 (α-linolenic acid)	0.32 (0.24–0.42)	0.30 (0.22–0.38)	0.32 (0.26–0.42)
C20:5n-3 (eicosapentaenoic acid)	0.87 (0.61–1.10)	0.80 (0.63–0.96)	0.78 (0.67–0.90)
C22:5n-3 (docosapentaenoic acid n-3)	0.73 (0.56–0.91)	0.70 (0.39–0.87)	0.81 (0.58–0.92)
C22:6n-3 (docosahexaenoic acid)	1.98 ^‡^ (1.42–2.69)	2.15 ^‡^ (1.22–2.84)	3.03 (2.62–3.73)
**Ratios**			
n-6/n-3	7.60 * (6.07–9.57)	7.97 (5.93–10.19)	6.66 (6.13–7.71)
C18:3n-6/C18:2n-6	0.018 ^‡^ (0.011–0.031)	0.028 ^‡^ (0.021–0.044)	0.008 (0.005–0.010)
C20:4n-6/C20:3n-6	2.54 ^‡^ (2.03–3.12)	2.56 ^‡^ (2.03–3.19)	3.42 (2.92–4.30)
C22:5n-6/C22:4n-6	0.910 ^†^ (0.738–1.071)	0.946 (0.706–1.321)	1.087 (0.896–1.330)
C22:6n-3/C22:5n-3	2.70 ^‡^ (2.05–3.68)	3.04 ^†^ (2.41–3.90)	3.88 (2.96–5.18)
C20:4n-6/C18:2n-6	0.42 (0.34–0.51)	0.45 (0.32–0.54)	0.42 (0.37–0.47)
C22:5n-6/C18:2n-6	0.014 (0.010–0.022)	0.019 ^†^ (0.009–0.032)	0.013 (0.010–0.017)
C20:5n-3/C18:3n-3	2.52 (1.79–3.54)	2.30 (1.78–3.71)	2.53 (2.03–3.01)
C22:6n-3/C18:3n-3	5.92 ^‡^ (4.21–8.94)	5.78 ^†^ (4.33–9.40)	9.17 (6.34–12.84)

Symbols indicate statistical significance in comparison against the control group (HS; * *p* < 0.05; ^†^ *p* < 0.01; ^‡^ *p* < 0.001).

**Table 4:** Fatty acid profile of serum glycerophospholipids in subgroups of cystic fibrosis (CF) patients defined by different clinical parameters.

	Age		Weight *Z-Score*		Height *Z-Score*		*CFTR* Genotype		Exocrine Pancreatic Insufficiency		Liver Disease		Diabetes		*P. aeruginosa*	
Median (1st–3rd quartiles)	<18	>18	≤−2	>−2	≤−2	>−2	Severe/severe	Other	Yes	No	Yes	No	Yes	No	Yes	No
*n*	77	95	23	149	28	144	110	62	148	24	79	93	25	147	88	84
C14:0	0.60 (0.51–0.73)	0.62 (0.49–0.82)	0.67 (0.56–0.83)	0.60 (0.48–0.76)	0.68 * (0.57–0.86)	0.58 * (0.47–0.76)	0.62 (0.52–0.78)	0.57 (0.44–0.77)	0.62 (0.50–0.79)	0.56 (0.43–0.67)	0.64 * (0.54–0.82)	0.57 * (0.47–0.73)	0.57 (0.51–0.81)	0.61 (0.49–0.76)	0.62 (0.52–0.79)	0.58 (0.48–0.76)
C16:0	30.74 (29.22–32.24)	31.48 (29.27–32.88)	31.71 (28.85–33.28)	31.06 (29.31–32.60)	31.37 (29.30–32.61)	30.94 (29.23–32.70)	31.20 (29.58–32.82)	30.04 (28.90–32.48)	31.10 (29.34–32.86)	31.01 (29.01–32.24)	31.29 * (29.41–33.44)	30.58 * (29.18–32.49)	31.98 (29.15–33.45)	30.98 (29.32–32.57)	31.14 (29.23–32.87)	30.98 (29.31–32.56)
C18:0	17.47 ^†^ (15.25–19.91)	15.87 ^†^ (14.64–17.63)	17.56 (15.46–18.56)	16.17 (14.64–19.16)	16.90 (15.68–18.55)	16.26 (14.66–18.94)	16.80 * (14.84–19.42)	15.83 * (14.65–17.30)	16.34 (14.59–18.88)	16.20 (15.25–19.42)	16.13 (14.78–18.75)	16.43 (14.78–19.30)	16.17 (14.78–17.76)	16.34 (14.75–19.23)	15.83 * (14.09–18.69)	16.81 * (15.44–19.44)
C16:1n-7	1.04 (0.78–1.42)	1.03 (0.75–1.43)	1.12 (0.73–1.47)	1.02 (0.77–1.42)	1.11 (0.79–1.66)	1.01 (0.76–1.42)	1.08 (0.78–1.46)	0.96 (0.75–1.26)	1.07 ^†^ (0.79–1.48)	0.83 ^†^ (0.67–0.98)	1.12 ^†^ (0.85–1.68)	0.94 ^†^ (0.70–1.28)	1.12 * (0.97–1.81)	1.01 * (0.72–1.42)	1.13 (0.76–1.62)	0.97 (0.76–1.28)
C18:1n-9	12.59 (10.73–14.86)	13.44 (12.29–14.61)	13.32 (10.45–16.85)	13.22 (12.08–14.60)	12.97 (12.09–15.55)	13.22 (11.79–14.61)	13.12 (11.66–14.77)	13.48 (12.21–14.65)	13.23 (11.97–14.82)	12.50 (12.07–13.91)	13.32 (11.81–14.74)	12.98 (12.08–14.63)	13.50 (12.28–14.83)	13.22 (11.64–14.62)	13.40 (12.21–15.11)	13.03 (11.50–14.48)
C20:1n-9	0.22 (0.15–0.34)	0.17 (0.12–0.29)	0.19 (0.13–0.31)	0.20 (0.14–0.31)	0.21 (0.14–0.37)	0.19 (0.13–0.30)	0.20 (0.14–0.34)	0.20 (0.13–0.30)	0.20 (0.13–0.33)	0.17 (0.14–0.23)	0.27 (0.21–0.34)	0.21 (0.14–0.34)	0.19 (0.11–0.33)	0.20 (0.14–0.31)	0.20 (0.14–0.30)	0.17 (0.13–0.33)
C20:3n-9	0.53 ^†^ (0.29–1.27)	0.35 ^†^ (0.24–0.58)	0.67 (0.34–1.11)	0.37 (0.25–0.76)	0.73 * (0.34–1.26)	0.36 * (0.25–0.76)	0.46 * (0.28–0.90)	0.33 * (0.23–0.58)	0.43 ^†^ (0.28–0.87)	0.25 ^†^ (0.19–0.42)	0.45 (0.29–0.83)	0.35 (0.25–0.86)	0.45 (0.33–0.67)	0.37 (0.25–0.86)	0.45 (0.29–0.80)	0.33 (0.25–0.88)
C18:2n-6	18.49 (14.98–21.00)	18.64 (16.39–20.23)	19.37 (16.73–21.53)	18.63 (16.04–20.37)	19.60 (15.40–20.91)	18.51 (16.09–20.38)	17.85 * (15.06–20.34)	19.13 * (17.75–21.00)	18.38 * (15.69–20.27)	20.10 * (18.42–21.06)	18.63 (15.76–19.84)	18.64 (16.23–21.00)	19.13 (16.61–19.56)	18.53 (15.85–20.52)	18.70 (16.42–20.30)	18.46 (15.88–20.73)
C18:3n-6	0.48 ^‡^ (0.27–0.65)	0.26 ^‡^ (0.18–0.38)	0.42 * (0.30–0.64)	0.29 * (0.20–0.510	0.48 ^†^ (0.30–0.66)	0.28 ^†^ (0.20–0.49)	0.34 (0.21–0.52)	0.28 (0.19–0.50)	0.33 (0.21–0.52)	0.27 (0.19–0.52)	0.34 (0.21–0.51)	0.28 (0.20–0.53)	0.30 (0.20–0.52)	0.32 (0.21–0.52)	0.35 (0.21–0.52)	0.28 (0.19–0.51)
C20:2n-6	0.29 (0.24–0.34)	0.28 (0.21–0.34)	0.24 * (0.20–0.29)	0.29 * (0.23–0.34)	0.25 (0.20–0.32)	0.29 (0.23–0.34)	0.28 (0.22–0.34)	0.29 (0.23–0.34)	0.28 (0.21–0.34)	0.28 (0.24–0.31)	0.27 (0.21–0.34)	0.28 (0.23–0.34)	0.25 * (0.18–0.34)	0.29 * (0.23–0.34)	0.28 (0.21–0.34)	0.28 (0.23–0.34)
C20:3n-6	3.12 * (2.61–3.81)	2.73 * (2.24–3.49)	2.88 (2.44–3.41)	3.00 (2.37–3.68)	2.81 (2.34–3.49)	3.02 (2.39–3.68)	3.03 (2.43–3.68)	2.88 (2.25–3.37)	3.03 (2.41–3.68)	2.67 (2.21–3.05)	3.02 (2.36–3.69)	2.93 (2.39–3.57)	2.76 (2.11–3.37)	3.03 (2.40–3.69)	2.89 (2.39–3.66)	3.02 (2.37–3.58)
C20:4n-6	7.57 (6.50–9.02)	8.08 (6.61–8.88)	6.84 * (5.78–7.99)	8.03 * (6.68–9.02)	6.66 † (5.77–7.65)	8.08 † (6.84–9.14)	7.53 * (6.49–8.66)	8.47 * (6.86–9.66)	7.77 (6.57–8.86)	8.33 (6.36–9.60)	7.47 * (6.20–8.59)	8.20 * (6.68–9.57)	7.28 * (45.06–8.57)	7.89 * (6.68–9.07)	7.59 (6.45–8.76)	7.96 (6.89–9.50)
C22:4n-6	0.31 * (0.24–0.44)	0.27 * (0.21–0.37)	0.24 (0.21–0.35)	0.30 (0.22–0.40)	0.27 (0.21–0.34)	0.30 (0.22–0.41)	0.31 (0.22–0.42)	0.28 (0.21–0.35)	0.30 * (0.22–0.41)	0.26 * (0.20–0.31)	0.30 (0.24–0.39)	0.30 (0.20–0.41)	0.24 (0.20–0.33)	0.30 (0.22–0.41)	0.30 (0.22–0.38)	0.29 (0.22–0.41)
C22:5n-6	0.34 ^‡^ (0.24–0.44)	0.23 ^‡^ (0.17–0.32)	0.23 (0.19–0.34)	0.27 (0.19–0.43)	0.23 (0.19–0.41)	0.26 (0.19–0.41)	0.28 (0.20–0.43)	0.24 (0.17–0.36)	0.27 † (0.19–0.44)	0.20 † (0.16–0.26)	0.26 (0.20–0.43)	0.25 (0.18–0.40)	0.23 (0.18–0.28)	0.27 (0.19–0.43)	0.24 (0.19–0.36)	0.29 (0.19–0.44)
C18:3n-3	0.30 (0.22–0.44)	0.33 (0.25–0.42)	0.29 (0.26–0.34)	0.33 (0.24–0.43)	0.31 (0.27–0.52)	0.32 (0.23–0.42)	0.31 (0.24–0.42)	0.33 (0.24–0.31)	0.31 (0.24–0.42)	0.31 (0.24–0.42)	0.31 (0.23–0.42)	0.33 (0.24–0.42)	0.32 (0.23–0.38)	0.32 (0.24–0.43)	0.33 (0.24–0.39)	0.31 (0.23–0.44)
C20:5n-3	0.86 (0.58–1.10)	0.87 (0.63–1.07)	0.69 (0.47–1.00)	0.87 (0.65–1.11)	0.73 (0.50–1.01)	0.88 (0.64–1.11)	0.91 (0.57–1.14)	0.83 (0.68–0.98)	0.88 (0.61–1.12)	0.81 (0.60–0.96)	0.81 (0.52–1.07)	0.91 (0.67–1.10)	0.70 (0.51–0.97)	0.88 (0.63–1.11)	0.81 (0.54–1.04)	0.91 (0.70–1.13)
C22:5n-3	0.74 (0.56–0.94)	0.68 (0.57–0.90)	0.51 † (0.39–0.78)	0.74 † (0.59–0.93)	0.66 (0.42–0.86)	0.74 (0.58–0.93)	0.76 (0.53–0.94)	0.67 (0.59–0.89)	0.74 (0.58–0.93)	0.61 (0.53–0.87)	0.72 (0.52–0.90)	0.73 (0.59–0.93)	0.60 * (0.44–0.78)	0.74 * (0.58–0.93)	0.71 (0.51–0.90)	0.73 (0.60–0.94)
C22:6n-3	2.02 (1.38–2.88)	1.91 (1.44–2.54)	1.51 * (1.23–2.09)	2.00 * (1.53–2.71)	1.65 (1.24–2.29)	2.00 (1.54–2.72)	1.95 (1.37–2.51)	2.09 (1.55–2.83)	1.97 (1.42–2.70)	2.09 (1.51–2.59)	1.70 † (1.25–2.44)	2.12 † (1.64–2.78)	1.55 * (1.25–2.03)	2.01 * (1.52–2.70)	1.77 * (1.32–2.54)	2.04 * (1.63–2.72)

* *p* < 0.05; ^†^ *p* < 0.01; ^‡^ *p* < 0.001.

**Table 5:** A multiple regression analysis of various factors predictive of the fatty acids profile in serum glycerophospholipids.

*p* Model	Dependent Variable	Independent Variable	*P*	β
0.0161	C16:0	*CFTR* genotype	0.0068	−0.2806
		Sex	0.0268	−0.1768
0.0017	C18:2n-6	*CFTR* genotype	0.0173	0.2410
		Sex	0.0338	0.1656
		FEV1	0.0475	0.1889
0.0015	C18:3n-6	Age	0.0002	−0.3718
		Diabetes	0.0218	−0.2051
0.0176	C20:3n-6	Pancreatic insufficiency	0.0183	0.2377
0.0041	C20:4n-6	Age	0.0280	0.2191
0.0014	C22:5n-6	Liver disease	0.0065	−0.2296
		Pancreatic insufficiency	0.0227	0.2238
0.0346	C22:6n-3	Pancreatic insufficiency	0.0099	0.2622
		*CFTR* genotype	0.0072	0.2809

*CFTR:* CF transmembrane conductance regulator.
